# Supplementary material for: Molecular Analysis of SARS-CoV-2 Genetic Lineages in Jordan: Tracking the Introduction and Spread of COVID-19 UK Variant of Concern at a Country Level
Source: Pathogens. 2021 Mar 5;10(3):302. doi: 10.3390/pathogens10030302 (PMC8000902; doi:10.3390/pathogens10030302)

Supplementary S1

Detailed maximum likelihood phylogenetic trees for the Jordanian SARS-CoV-2 sequences

Maximum likelihood (ML) phylogenetic trees constructed using SARS-CoV-2 sequences collected in Jordan transformed into cladograms are shown in this supplementary. The first ML tree was constructed using *ORF1ab* region, while the second ML tree was constructed using the *Spike* region. Internal branches with approximate likelihood Shimodaira-Hasegawa (aLRT-SH) values of ≥ 0.90 are shown in red. The clustered first Jordan lineage (B.1.1.312) sequences are shown as purple terminal branches; the clustered second Jordan lineage (B.1.36.10) are shown as green terminal branches; and the clustered UK variant lineage (B.1.1.7) are shown as blue terminal branches; ORF: open reading frame.

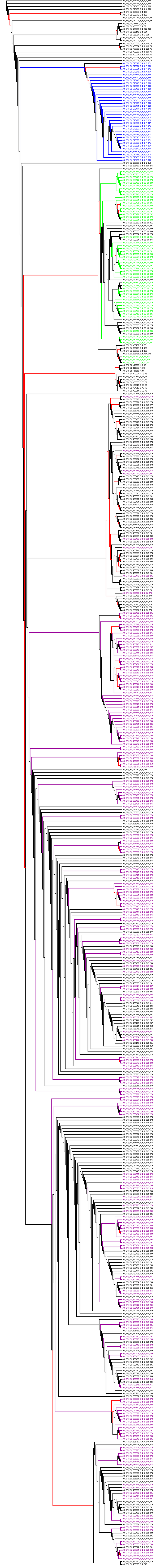

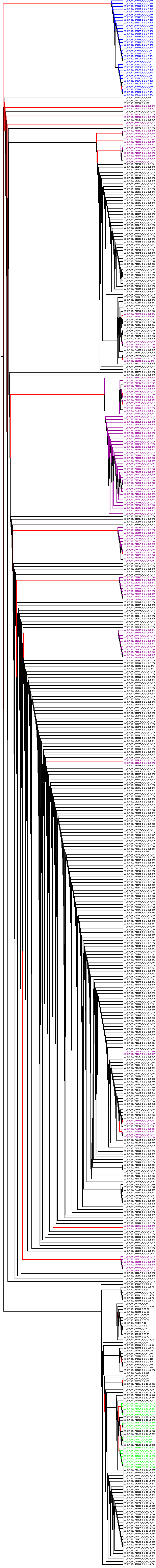

Supplement: Supplementary file 1 [file pathogens-10-00302-s001.pdf]
